# Deficiency of CCAAT/enhancer‐binding protein homologous protein (CHOP) prevents diet‐induced aortic valve calcification *in vivo*

**DOI:** 10.1111/acel.12674

**Published:** 2017-09-10

**Authors:** Zhejun Cai, Baoqing Liu, Jia Wei, Zurong Fu, Yidong Wang, Yaping Wang, Jian Shen, Liangliang Jia, Shengan Su, Xiaoya Wang, Xiaoping Lin, Han Chen, Fei Li, Jian'an Wang, Meixiang Xiang

**Affiliations:** ^1^ Department of Cardiology Second Affiliated Hospital, Zhejiang University School of Medicine Hangzhou China; ^2^ Department of Cardiovascular Surgery Union Hospital, Tongji Medical College, Huazhong University of Science and Technology Wuhan China; ^3^ Department of Urology Children's Hospital, Zhejiang University School of Medicine Hangzhou China

**Keywords:** aortic valve calcification, apoptotic bodies, apoptosis, CHOP, endoplasmic reticulum stress

## Abstract

Aortic valve (AoV) calcification is common in aged populations. Its subsequent aortic stenosis has been linked with increased morbidity, but still has no effective pharmacological intervention. Our previous data show endoplasmic reticulum (ER) stress is involved in AoV calcification. Here, we investigated whether deficiency of ER stress downstream effector CCAAT/enhancer‐binding protein homology protein (CHOP) may prevent development of AoV calcification. AoV calcification was evaluated in Apoe^−/−^ mice (*n* = 10) or in mice with dual deficiencies of ApoE and CHOP (Apoe^−/−^ CHOP ^−/−^, *n* = 10) fed with Western diet for 24 weeks. Histological and echocardiographic analysis showed that genetic ablation of CHOP attenuated AoV calcification, pro‐calcification signaling activation, and apoptosis in the leaflets of Apoe^−/−^ mice. In cultured human aortic valvular interstitial cells (VIC), we found oxidized low‐density lipoprotein (oxLDL) promoted apoptosis and osteoblastic differentiation of VIC via CHOP activation. Using conditioned media (CM) from oxLDL‐treated VIC, we further identified that oxLDL triggered osteoblastic differentiation of VIC via paracrine pathway, while depletion of apoptotic bodies (ABs) in CM suppressed the effect. CM from oxLDL‐exposed CHOP‐silenced cells prevented osteoblastic differentiation of VIC, while depletion of ABs did not further enhance this protective effect. Overall, our study indicates that CHOP deficiency protects against Western diet‐induced AoV calcification in Apoe^−/−^ mice. CHOP deficiency prevents oxLDL‐induced VIC osteoblastic differentiation via preventing VIC‐derived ABs releasing.

## Introduction

Calcified aortic valve disease (CAVD) is the most common valvular disease in the Western World (Nkomo *et al*., 2006). CAVD may finally develop into aortic valve (AoV) stenosis. Calcified AoV stenosis is associated with increased cardiovascular events and mortality and is present in > 4% of very elderly patients (Iung *et al*., 2003; Nkomo *et al*., 2006). So far, there is no effective pharmacological therapy for CAVD other than surgical or interventional AoV replacement (Lerman *et al*., 2015). In the past decades, accumulating evidences suggest that AoV calcification is not a passive degenerative process, but an active disease progress including lipoprotein deposition, chronic inflammation, and osteoblastic differentiation of valvular interstitial cells (VIC) (Mathieu *et al*., 2014; Lerman *et al*., 2015).

The endoplasmic reticulum (ER) is a crucial multifunctional organelle that regulates a wide range of cellular process including protein synthesis, folding, and transportation (Ron & Walter, 2007). Disruption of ER homeostasis causes accumulation of unfolded and misfolded proteins in the ER lumen, leading to activation of ER stress signaling. ER stress signaling is composed of three signaling axes that are initiated by inositol‐requiring protein‐1 (IRE1), double‐stranded RNA‐dependent protein kinase‐like ER kinase (PERK), and activating transcription factor 6 (ATF6)(Hetz, 2012). Numerous studies have shown that excessive ER stress induction is linked with a variety of diseases, such as diabetes mellitus (Ozcan *et al*., 2006), obesity (Ozcan *et al*., 2004), and atherosclerosis (Myoishi *et al*., 2007). Our previous study also indicates that ER stress participates in the development of AoV calcification (Cai *et al*., 2013).

Among the three branches of ER stress signaling, activation of the PERK pathway induces the CCAAT/enhancer‐binding protein homology protein (CHOP). CHOP activation promotes apoptosis and is involved the pathogenesis of vascular diseases such as atherosclerosis (Zhou *et al*., 2015), chronic kidney disease‐induced vascular medial calcification (Miyazaki‐Anzai *et al*., 2014). Our previous report also found CHOP expression is high in calcified AoV (Cai *et al*., 2013), and apoptosis of VIC has been reported to be essential in development of AoV calcification (Jian *et al*., 2003; Galeone *et al*., 2013). These indicate that CHOP may also promote apoptosis of VIC, and finally lead to AoV calcification.

In this study, we aimed to investigate the contribution of CHOP in diet‐induced AoV calcification. In addition, we explored the role of CHOP in osteoblastic differentiation of VIC associated with AoV calcification.

## Results

### Deficiency of CHOP prevents diet‐induced AoV calcification in Apoe^−/−^ mice

**Figure 1:**
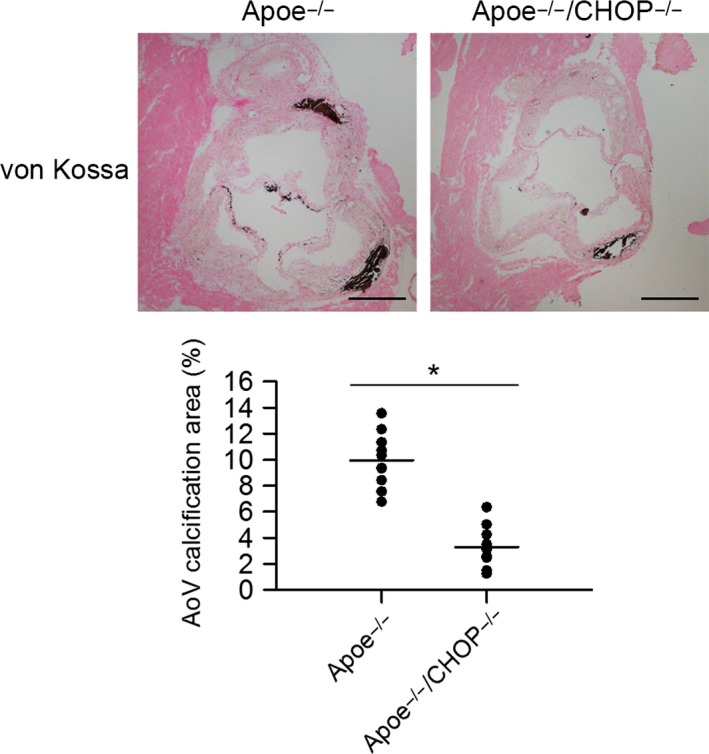
CHOP deficiency attenuates diet‐induced AoV calcification area in Apoe^−/−^ mice. Representative von Kossa staining of the AoV leaflets of Apoe^−/−^ mice and Apoe^−/−^ CHOP ^−/−^ mice challenged with Western diet for 24 weeks. CHOP ablation significantly reduced leaflet calcification area in Apoe^−/−^ mice. (Scale bar = 500 μm. *n* = 10 for each group. *P <* *0.05).

Our previous study found CHOP was activated in AoV calcification (Cai *et al*., 2013), here we investigated whether CHOP plays a role in AoV calcification *in vivo*. Mice in Apoe^−/−^ background were subjected to Western diet for 24 weeks to generate AoV calcification (Rajamannan, 2011). Von Kossa staining was applied to assess the calcium deposits in AoV leaflets. Immunofluorescence staining was applied to validate the efficacy of CHOP ablation in AoV leaflets of Apoe^−/−^CHOP^−/−^ mice (Fig. S1, Supporting Information). As expected, compared to Apoe^−/−^ mice, Apoe^−/−^CHOP^−/−^ mice exhibited significantly reduced AoV calcium deposits (Fig. 1). CHOP deficiency also reduced AoV leaflet thickness compared to Apoe^−/−^ mice (Fig. S2, Supporting Information).

We further detected the hemodynamic parameters by ultrasound. Paralleled with reduced calcium deposits, CHOP deletion led to decreased transvalvular peak jet velocity compared to Apoe^−/−^ mice (Fig. S2, Supporting Information). No significant difference of left ventricular function was observed between groups (Table S1, Supporting Information).

The metabolic parameters were compared. CHOP deficiency did not change the lipid profiles or glucose levels (Table S2, Supporting Information). Therefore, CHOP deficiency mitigates Western diet‐induced AoV calcification in ApoE^−/−^ mice without altering lipid and glucose metabolism.

### Ablation of CHOP inhibits apoptosis in AoV leaflets in Apoe^−/−^ mice

**Figure 2:**
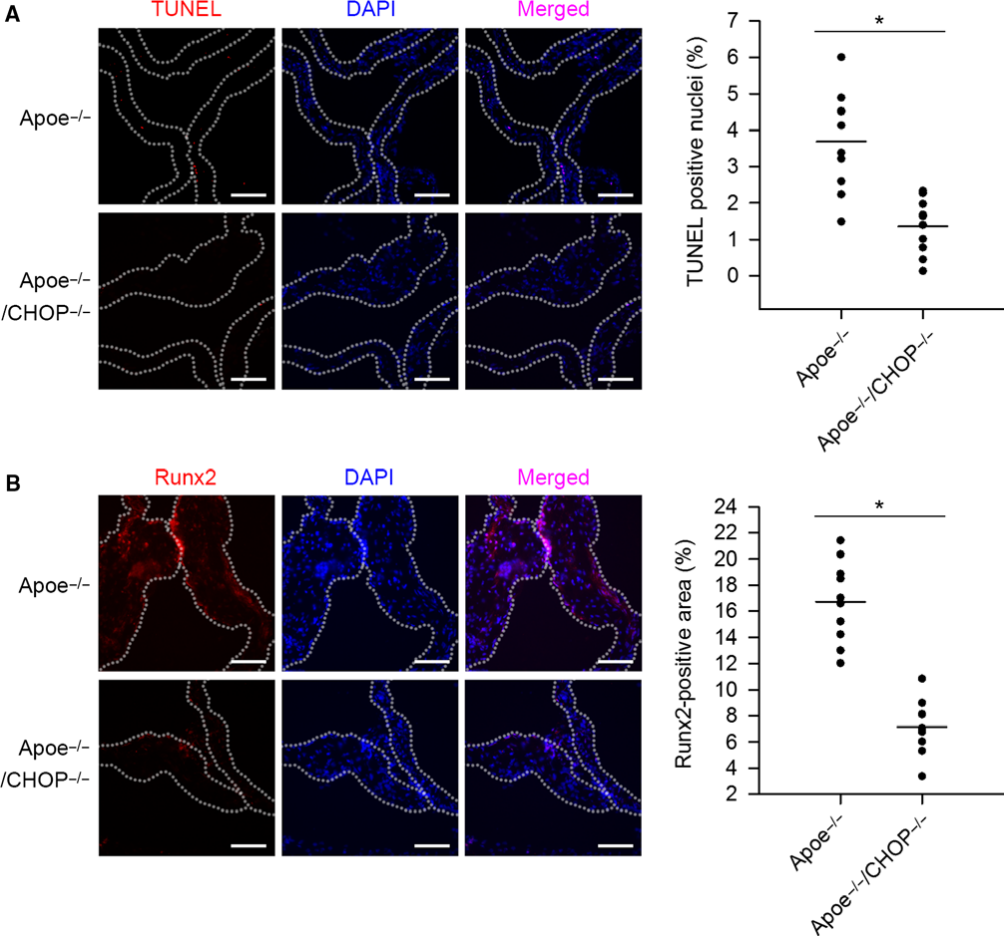
Ablation of CHOP suppresses leaflet apoptosis and pro‐calcification signaling activation in Apoe^−/−^ mice. (A) Representative TUNEL staining of AoV leaflets of Apoe^−/−^ mice and Apoe^−/−^ CHOP ^−/−^ mice. CHOP deficiency significantly suppressed leaflet apoptosis in Apoe^−/−^ mice. (B) Representative immunofluorescence staining of Runx2 of AoV leaflets of Apoe^−/−^ mice and Apoe^−/−^ CHOP ^−/−^ mice. CHOP deficiency significantly reduced leaflet Runx2 expression in Apoe^−/−^ mice. (Scale bar = 100 μm. *n* = 10 for each group. *P <* *0.05).

Studies indicate the role of apoptosis in the pathogenesis of AoV calcification (Galeone *et al*., 2013). We examined the effect of CHOP deficiency on apoptosis in AoV leaflets. As shown in Fig. 2A, CHOP deficiency in Apoe^−/−^ mice significantly reduced terminal deoxynucleotidyl transferase‐mediated dUTP nick end‐labeling (TUNEL) staining in AoV leaflets compared to Apoe^−/−^ mice. Moreover, CHOP ablation also significantly suppressed cleaved caspase‐3 expression in the AoV leaflets (Fig. S3, Supporting Information). These results suggest that CHOP ablation inhibits apoptosis in AoV leaflets.

Increased apoptosis indicates enhanced production of apoptotic bodies (ABs). It has been reported that phagocytosis of ABs is mainly mediated by macrophages (Takemura *et al*., 2007), we checked whether CHOP deficiency may affect ABs clearance by macrophages. In Fig. S3 (Supporting Information), although CHOP deficiency reduced cleaved caspase‐3 expression in the AoV leaflet, the percentage of CD68‐merged (macrophage marker) cleaved caspase‐3 was unchanged compared to Apoe^−/−^ mice.

### CHOP ablation suppresses pro‐osteogenic signaling activation in AoV leaflets in Apoe^−/−^ mice

We further tested the pro‐calcification signaling. Compared to Apoe^−/−^ group, Apoe^−/−^CHOP^−/−^ mice exhibited significantly reduced runt related transcription factor 2 (Runx2) staining in AoV leaflets (Fig. 2B). In addition, we found CHOP deficiency decreased Runx2, osteocalcin, and alkaline phosphatase (ALP) mRNA expression in AoV leaflets compared to Apoe^−/−^ mice (Fig. S4, Supporting Information). Collectively, these results demonstrate that CHOP deficiency inhibits pro‐calcification signaling in AoV leaflets.

### OxLDL‐induced osteoblastic differentiation of VIC via CHOP activation

**Figure 3:**
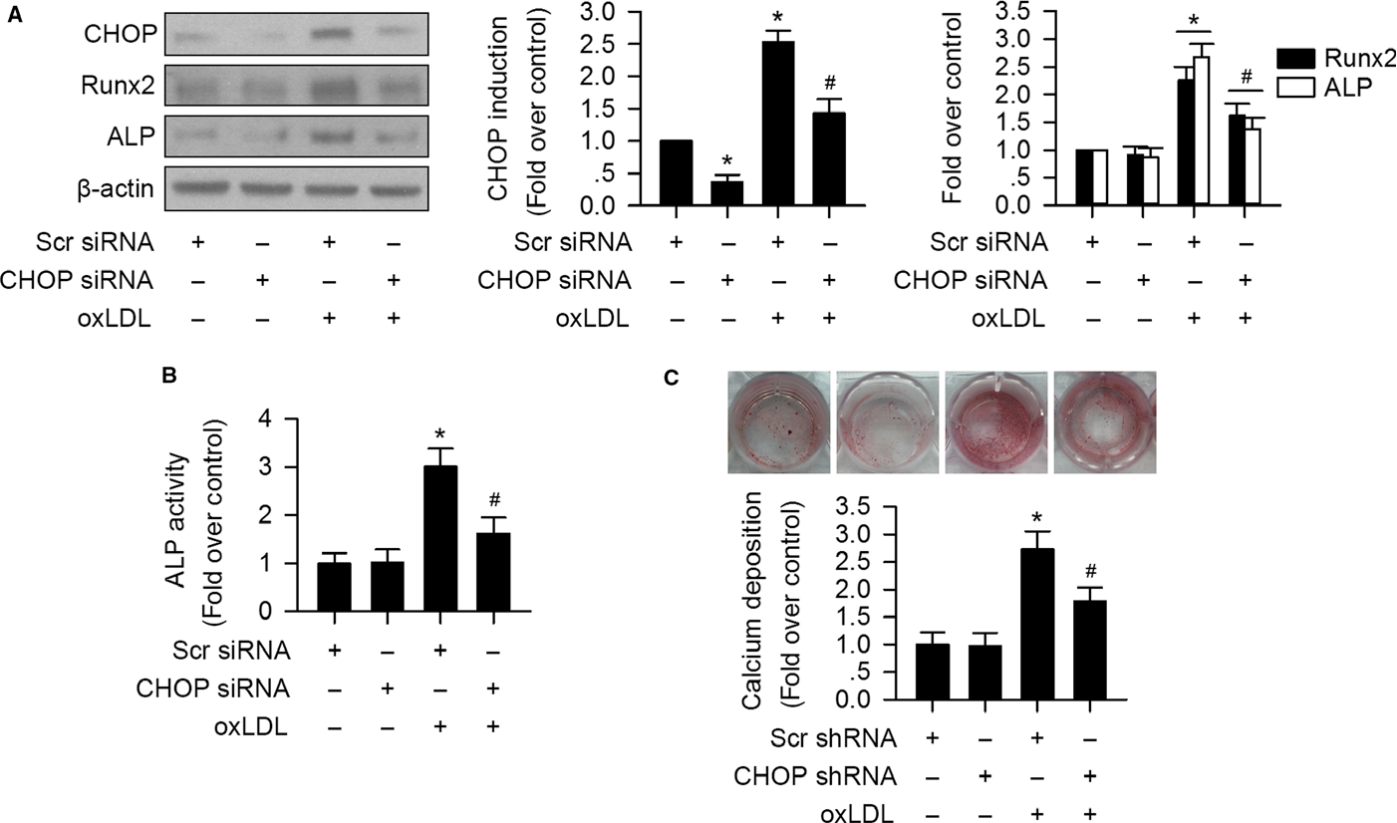
OxLDL promotes osteoblastic differentiation of VIC through CHOP activation. (A) OxLDL significantly increased CHOP expression accompanied with increased Runx2 and ALP expression in VIC. CHOP siRNA silencing significantly suppressed oxLDL‐induced Runx2 and ALP expression in VIC. (B) Silencing of CHOP remarkedly reduced oxLDL‐induced ALP activity in VIC. (C) Alizarin red staining of calcification nodules of VIC. Knock down of CHOP significantly reduced oxLDL‐induced calcium deposits of VIC. (*n* = 3 for each experiment; *P <* *0.05 vs. scrambled siRNA or shRNA served as control [Scr siRNA or Scr shRNA]; ^#^ *P *<* *0.05 vs. oxLDL + Scr siRNA or Scr shRNA).

OxLDL has been proved to play an essential role in AoV calcification development (Syvaranta *et al*., 2014). We determined whether CHOP is involved in oxLDL‐induced osteoblastic differentiation of human VIC. Here, we found that oxLDL induced CHOP as well as pro‐calcification signaling Runx2 and ALP expression in VIC (Fig. 3A). However, CHOP silencing significantly reduced oxLDL‐induced Runx2 expression in VIC (Fig. 3A). In addition, CHOP silencing effectively blocked ALP activity elevation induced by oxLDL (Fig. 3B).

To further confirm the role of CHOP in osteoblastic differentiation of VIC, lentiviral shRNA targeting CHOP was introduced. The knockdown efficacy was validated 14 days after lentiviral shRNA transfection (Fig. S5, Supporting Information). As shown in Fig. 3C, oxLDL treatment for 14 days significantly induced calcium deposits assessed by alizarin red staining, which was attenuated by CHOP silencing.

We further tested whether CHOP affects oxLDL uptake in VIC. As depicted in Fig. S6 (Supporting Information), silencing of CHOP did not alter uptake of Dil‐oxLDL.

Taken together, these data suggest that CHOP mediates oxLDL‐induced osteoblastic differentiation in VIC, which is independent of affecting oxLDL uptake.

### Suppression of CHOP prevents oxLDL‐induced VIC apoptosis

**Figure 4:**
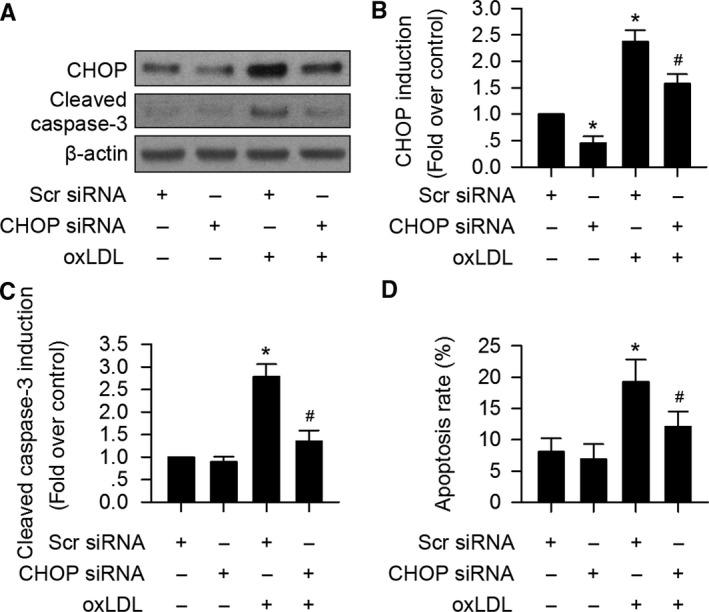
OxLDL induces VIC apoptosis via CHOP. (A–C) OxLDL significantly increased cleaved caspase‐3 and CHOP expression in VIC. CHOP siRNA silencing markedly suppressed oxLDL‐induced cleaved caspase‐3 expression in VIC. (D) Flowcytometry analysis of Annexin/PI double labeled VIC showed that CHOP silencing significantly prevented oxLDL‐mediated VIC apoptosis. (*n* = 3 for each experiment; *P <* *0.05 vs. Scr siRNA served as control; ^#^ *P *<* *0.05 vs. oxLDL + Scr siRNA).

CHOP has been shown to be a pro‐apoptotic protein (Dai *et al*., 2016). We next assayed whether CHOP also mediates oxLDL‐induced apoptosis of VIC. As expected, oxLDL significantly triggered cleaved caspase‐3 expression, while CHOP silencing markedly suppressed the effect (Fig. 4A–C). The Annexin V/PI double staining further confirmed that CHOP silencing suppressed oxLDL‐induced VIC apoptosis (Fig. 4D).

### CHOP promotes oxLDL‐induced VIC osteoblastic differentiation in paracrine pathway

**Figure 5:**
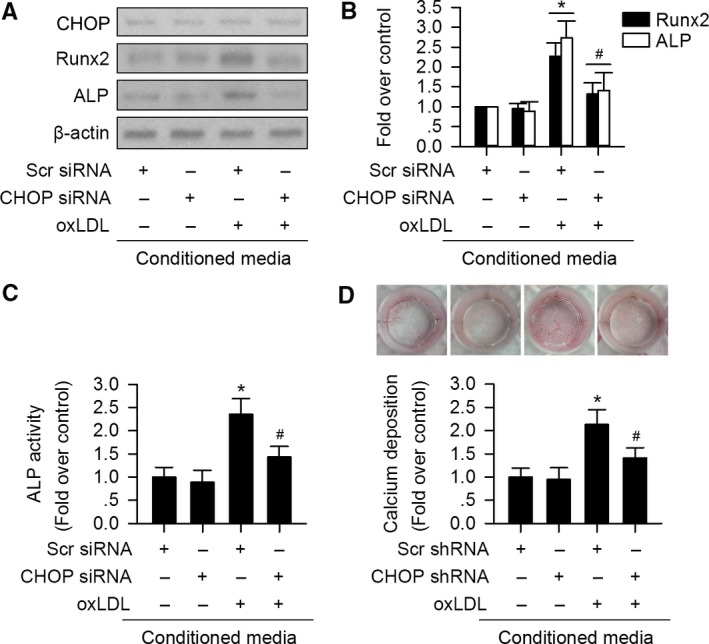
Paracrine is involved in oxLDL/CHOP signaling‐induced osteoblastic differentiation of VIC. Conditioned media (CM) from oxLDL‐treated control VIC significantly increased Runx2 and ALP expression (A, B) as well as ALP activity of VIC without altering CHOP expression (C). However, CM from oxLDL‐treated CHOP‐silenced VIC had significantly reduced effects (A–C). (D) Alizarin red staining of calcification nodules of VIC. CM from oxLDL‐incubated control VIC promoted calcium deposits of VIC, but CM from oxLDL‐incubated CHOP‐silenced VIC suppressed the effect. (*n* = 3 for each experiment; *P <* *0.05 vs. Scr siRNA or Scr shRNA served as control; ^#^ *P *<* *0.05 vs. oxLDL + Scr siRNA or Scr shRNA).

We compared the effect of conditioned media (CM) from oxLDL‐treated VIC on osteoblastic differentiation. As shown in Fig. 5A–C, compared to CM from vehicle‐incubated cells, CM from oxLDL‐incubated VIC significantly promoted osteogenic proteins ALP and Runx2 expression as well as ALP activity in VIC. However, the effects were significantly blocked when VIC was challenged with CM from oxLDL‐treated CHOP‐silenced cells without altering CHOP expression (Fig. 5A–C).

We further compared the osteoblastic differentiation of VIC. CM from oxLDL‐treated VIC significantly promoted calcium deposits in VIC compared to CM from vehicle‐incubated VIC (Fig. 5D). As expected, VIC treated with CM from oxLDL‐incubated CHOP‐silenced cell had less calcium deposits compared to cells treated with CM from oxLDL‐incubated scramble shRNA‐transfected cells (Fig. 5D).

These data indicate that CHOP mediates oxLDL‐promoted VIC osteoblastic differentiation in paracrine pathway.

### The preventive effect of CHOP deficiency on VIC osteoblastic differentiation is mediated by VIC‐derived ABs

Our data imply that CHOP promotes oxLDL‐induced apoptosis, and the paracrine pathway participates in oxLDL/CHOP‐mediated VIC osteoblastic differentiation. We next assayed whether ABs released by VIC are involved in the process.

**Figure 6:**
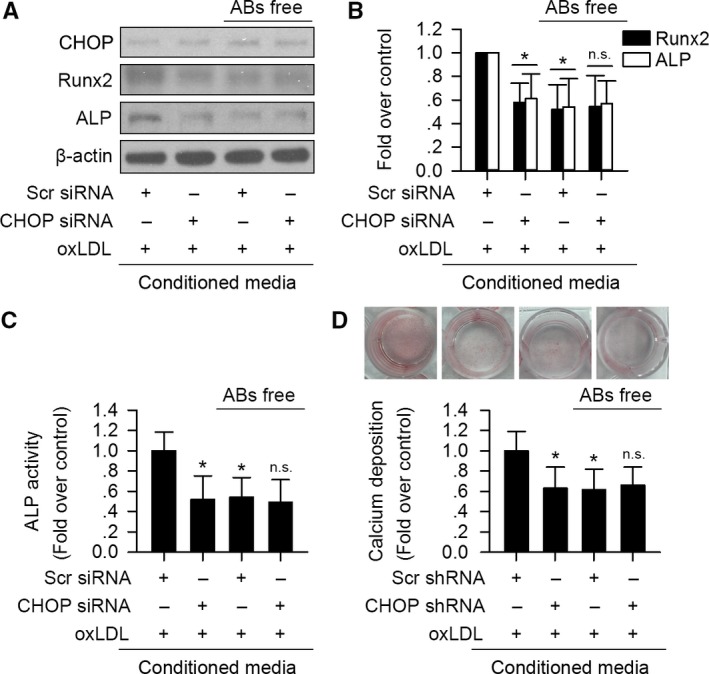
Apoptotic bodies mediate oxLDL/CHOP signaling‐induced osteoblastic differentiation of VIC. VIC treated with conditioned media (CM) from oxLDL‐exposed CHOP‐silenced VIC had significantly reduced Runx2 and ALP expression (A, B), ALP activity (C), and calcium deposits (D) without altering CHOP expression, compared to treated with CM from oxLDL‐exposed control VIC. Depletion of apoptotic bodies (ABs) in CM significantly suppressed oxLDL‐incubated control VIC's CM‐induced Runx2 and ALP expression (A, B), ALP activity (C), and calcium deposits (D) without altering CHOP expression. ABs depletion in oxLDL‐incubated CHOP‐silenced VIC's CM did not further enhance these protective effects (A–D). There was no significant difference of Runx2 and ALP expression, ALP activity, and calcium deposits between VIC treated with AB‐free oxLDL‐incubated control cells’ CM and AB‐free oxLDL‐incubated CHOP‐silenced cells’ CM (A–D). (*n* = 3 for each experiment; *P <* *0.05 vs. oxLDL + Scr siRNA or Scr shRNA; n.s. no significant difference vs. oxLDL + CHOP siRNA or shRNA and AB‐free oxLDL + Scr siRNA or Scr shRNA).

Addition of ABs elevated ALP activity and promoted calcium deposits of VIC (Fig. S7, Supporting Information). As depicted in Fig. 6, compared to oxLDL‐treated VIC's CM, VIC treated with AB‐free CM from oxLDL‐incubated cells suppressed osteogenic mediators Runx2 and ALP expression, as well as ALP activity and calcium deposits without altering CHOP expression. Although VIC incubated with CM from oxLDL‐incubated CHOP‐silenced cells had reduced Runx2 and ALP expression, ALP activity, and calcium deposits, free of AB did not further promote the protective effects (Fig. 6). In addition, there was no significant difference of Runx2 and ALP expression, ALP activity, and calcium deposits between VIC treated with AB‐free oxLDL‐incubated control cells’ CM and AB‐free oxLDL‐incubated CHOP‐silenced cells’ CM (Fig. 6).

Taken together, these data further suggest that VIC‐derived ABs mediate oxLDL/CHOP signaling‐induced osteoblastic differentiation of VIC.

## Discussion

Despite the clinical importance of CAVD, the underlying mechanisms still need to be explored. The present study expands our previous work and suggests the important role of CHOP in the pathogenesis of AoV calcification. Here, we show that CHOP deficiency attenuates the development of diet‐induced AoV calcification in Apoe^−/−^ mice, which is associated with reduced apoptosis and pro‐calcification signaling activation in the AoV leaflets. Our *in vitro* data indicate that oxLDL promotes VIC apoptosis and osteoblastic differentiation of VIC via CHOP activation. The pro‐osteogenic effect of oxLDL is mediated, at least in part, by paracrine pathway, and CHOP participates in the process. Furthermore, we identify that the protective effects of CHOP deficiency on suppressing osteoblastic differentiation of VIC are mediated by ABs.

The most important finding of the study is that deficiency of CHOP suppressed diet‐induced AoV calcification in Apoe^−/−^ mice. The role of CHOP in vascular diseases, such as atherosclerosis, has been widely documented (Zhou *et al*., 2015). One recent study suggests that CHOP deficiency prevents chronic kidney disease‐dependent vascular calcification *in vivo* (Miyazaki‐Anzai *et al*., 2014). In our previous work, we observed increased CHOP expression in calcified AoV leaflets, and oxLDL induces CHOP upregulation in VIC (Cai *et al*., 2013). These evidences suggest that CHOP may also participate in development of AoV calcification. Indeed, we directly confirm the role of CHOP in AoV calcification, that CHOP deficiency in Apoe^−/−^ mice exhibited reduced pro‐osteogenic markers expression and calcium deposition in AoV leaflets. These results demonstrate that CHOP may act as a therapeutic target for AoV calcification.

It has been widely reported that CHOP activation leads to apoptosis (Nishitoh, 2012), while apoptosis of VIC is linked with development of AoV calcification (Galeone *et al*., 2013). It is possible that CHOP ablation may prevent AoV calcification via suppressing apoptosis. Indeed, we confirm that CHOP deficiency reduces oxLDL‐induced apoptosis of VIC and apoptosis in AoV leaflets of Western diet‐fed mice.

Another key finding of this work is that CHOP deficiency prevents oxLDL‐induced osteoblastic differentiation of VIC is mediated by ABs. Our *in vivo* data show CHOP deficiency reduces apoptosis in AoV leaflets, indicating reduced ABs production. Proudfoot *et al*. (2000) reported that ABs mediate vascular calcification. We hypothesized that ABs may also mediate osteoblastic differentiation of VIC. Our data show that oxLDL triggers osteoblastic differentiation of VIC in paracrine pathway, and most importantly, ABs mediate oxLDL‐induced osteoblastic differentiation of VIC. We further found the CM from oxLDL‐treated CHOP‐silenced VIC suppressed osteoblastic differentiation of VIC, and ablation of ABs did not further enhance this protective effect, which strongly supports the protective effect of CHOP deficiency on osteoblastic differentiation of VIC is via ABs.

It has been reported that ABs contribute to vascular calcification (Proudfoot *et al*., 2000). Accumulation of ABs has been observed in advanced atherosclerotic plaques accompanied with microcalcification (Otsuka *et al*., 2015), and VSMC‐derived ABs can initiate calcification of VSMC (Proudfoot *et al*., 2000; Kapustin *et al*., 2015). Such ABs contain abundant calcium salts and cathepsin S (Proudfoot *et al*., 2000; Kapustin *et al*., 2011), which promote osteoblastic differentiation of VIC (Aikawa *et al*., 2009; Zeng *et al*., 2017). However, the components to promote calcification of VIC need to be further explored.

The clearance of ABs may also affect the effect of CHOP deficiency on AoV calcification. It has been documented that clearance of ABs is majorly mediated by macrophage phagocytosis (Takemura *et al*., 2007), but our results did not found alteration of macrophages phagocytosis of cleaved caspase‐3. However, our study did not detect the release of ABs in the blood, which may be a limitation of the work.

In the present study, CHOP deficiency does not fully block the progression of AoV calcification. This indicates that there exist pathways in parallel with CHOP, leading to AoV calcification, for example, Toll‐like receptors signaling (Yang *et al*., 2009), Wnt/Notch1 signaling (Nigam & Srivastava, 2009), peroxisome proliferator‐activated receptor‐γ signaling (Chu *et al*., 2013). It should be noted that besides apoptosis, proliferation of VIC is also involved in the pathogenesis of CAVD (Li *et al*., 2013). It has been documented that oxLDL could not only promote apoptosis (Larroque‐Cardoso *et al*., 2013) but also enhance proliferation of vascular smooth muscle cells (Lin *et al*., 2016). Whether oxLDL also promotes AoV calcification and CAVD by enhancing proliferation of VIC needs to be explored in future study.

To sum up, CHOP deficiency prevents diet‐induced AoV calcification *in vivo*, which is via preventing apoptosis and VIC‐derived ABs pathway. Combining with our previous findings that ER chemical chaperones suppress AoV calcification (Cai *et al*., 2013), our data suggest that the ER stress‐mediated CHOP signaling may be a potential therapeutic target for AoV calcification.

## Experimental procedures

### Materials

Antibodies against CHOP, Runx2, β‐actin, ALP, cleaved caspase‐3, and ALP were purchased from Cell Signaling Tech (Danvers, MA, USA). Antibody against CD68 was purchased from R&D system (Minneapolis, MN, USA). ALP assay kit and Von Kossa kit were purchased from Abcam (Cambridge, MA, USA). Calcium diagnostic kit was from Teco Diagnostic (Anaheim, CA, USA). Lentiviral shRNA particles and siRNA for CHOP were purchased from Santa Cruz Biotech, Inc (Dallas, TX, USA). Controls were the ‘scrambled’ shRNA expression construct or siRNA from Santa Cruz Biotech. RNAiso Plus, PrimeScript™ RT Master Mix, and SYBR_Premix Ex Taq™ were purchased from Takara Bio Inc (Kusatsu, Japan).

### Animals

All animal experimental protocols complied with the Guide for the Care and Use of Laboratory Animals published by the US National Institutes of Health (NIH Publication No. 85‐23, revised 1996). The animal studies were approved by the Institutional Animal Research Committee of Zhejiang University.

Apoe^−/−^ and CHOP^−/−^ mice (C57BL/6 background) were purchased from Model Animal Research Center of Nanjing University (Nanjing, China). Apoe^−/−^CHOP^−/−^ mice were generated by crossing Apoe^−/−^ mice with CHOP^−/−^ mice. The mice were housed in a controlled environment (20 ± 2 °C, 12‐h/12‐h light/dark cycle) and had free access to water and diet.

AoV calcification was generated by a 24‐week protocol as described previously (Cai *et al*., 2013). After initial quarantine, 12‐week old mice were assigned into two groups: Apoe^−/−^ mice receiving Western diet; Apoe^−/−^CHOP^−/−^ mice receiving Western diet. *N* = 10 for each group. At the end of the experiment, mice were sacrificed. Tissues and blood were collected.

### Echocardiography and tissue processing

At the end of the experiment, transthoracic echocardiography was performed under 2.5% isoflurane anesthesia. The images were acquired by Vevo 2100 Imaging system. The transvalvular velocity was evaluated via continuous wave Doppler. The echocardiographic data were collected by an experienced operator blinded to the assignments.

Following final transthoracic echocardiography, mice were sacrificed and blood was collected. Mice were then perfused via the left ventricle with 5 mL PBS prior to tissue collection. Heart with aortic roots was carefully dissected and embedded in optimum cutting temperature compound (OCT; BDH Laboratory Supplies, Safat, Kuwait).

### Von Kossa staining

Sections were cut at 8 μm for OCT‐embedded samples. Serial cryosections were collected and stained with von Kossa staining kit (Abcam) according to manufacturer's instruction (Cai *et al*., 2016).

### Immunofluorescence staining

For the immunofluorescence staining analysis, slides were stained with antibodies specific to Runx2. Olympus fluorescence microscope was used for images collecting. Image‐Pro Plus 6.0 (Media Cybernetics, Rockville, MD, USA) was applied for data quantification.

### TUNEL staining

Slides were stained with terminal deoxynucleotidyl transferase‐mediated dUTP nick end‐labeling (TUNEL) (In Situ Cell Death Detection Kit, TMR red; Roche Applied Science, Indianapolis, IN, USA) according to manufacturer's instruction.

### Determination of blood glucose, cholesterol, LDLs, and triglyceride levels

Blood glucose, cholesterol, triglyceride, calcium, and phosphorus levels were determined using indicated kits from Biosino, China, according to the manufacturer's instructions.

### Quantitative real‐time PCR

Total RNA was isolated from AoV tissues by Laser Capture Microdissection from the cryosections using PureLink RNA Micro Kit (Invitrogen, Waltham, MA, USA) according to the manufacturer's instructions. Reverse transcription of RNA was performed using PrimeScript™ RT Master Mix (Takara). Quantitative real‐time PCR was performed using SYBR Premix Ex TaqTM (Takara) on a StepOnePlusTM Real‐time PCR System (Applied Biosystems, Foster City, CA, USA). The primers for target genes were listed in Table S3 (Supporting Information). All PCRs were performed in duplicate, and mRNA fold changes were calculated by the 2^−ΔΔCt^ method using β‐actin as internal reference.

### Human aortic valve collection

This study complied with the Declaration of Helsinki and was approved by the Ethic Board of Second Affiliated Hospital, Zhejiang University School of Medicine. All patients provided written informed consent. The normal AoV leaflets were collected from dilated cardiomyopathy patients with normal aortic valves undergoing heart transplantation surgery. The demographic information of patients was summarized in Table S4 (Supporting Information).

### Cell culture

Human VIC were isolated from normal valves by collagenase I digestion method (Wang *et al*., 2017). Briefly, isolated leaflets were digested in medium containing 1 mg mL^−1^ collagenase I at 37 °C for 30 min. After vortex, the leaflets were further digested with a fresh solution of 1 mg mL^−1^ collagenase medium for 4–6 h at 37 °C. After repeated aspirating to break up the tissue mass, the suspension was spun at 800 g for 10 min to precipitate cells. Cells were re‐suspended and cultured in M199 growth medium, supplemented with 100 U mL^−1^ penicillin, 100 μg mL^−1^ streptomycin and 10% fetal bovine serum in an incubator with 5% CO_2_ at 37 °C. Cells of passage 2–6 were used for all experiments.

### Isolation of LDL and preparation of oxLDL

LDL (density = 1.019–1.063 g mL^−1^) were isolated from pooled human plasma by sequential ultracentrifugation as previously described (Cai *et al*., 2013). For oxidation, LDL was incubated with 10 mmol L^−1^ CuSO_4_ for 18 h at 37 °C and terminated by adding 1 mmol L^−1^ EDTA. OxLDL was then dialyzed, sterilized by filtering, and diluted to 1 mg mL^−1^ in PBS. The prepared oxLDL was stored at 4 °C and used within 2 weeks.

### Cell treatment

When the cells were grown to 80–90% confluence, serum‐starved overnight and treated with oxLDL (100 ng mL^−1^). VIC were treated for 48 h for detection of protein expression, ALP activity, and apoptosis.

### Preparation of conditioned media

Cells were treated accordingly for 48 h, washed with PBS twice. The media were then changed to fresh media, and cells were further incubated for another 12 h. The media were collected as the conditioned media.

### Cellular small‐interfering RNA transfection

Valvular interstitial cells were transfected with control small‐interfering RNA (siRNA), CHOP siRNA duplex (Santa Cruz Biotechnology), accordingly with RNAiMax (Life Technology, Grand Island, NY, USA).

### Lentivirus transfection

Valvular interstitial cells were infected with recombinant lentiviruses expressing control shRNA (Santa Cruz Biotechnology) and CHOP shRNA (Santa Cruz Biotechnology) according to manufacturer's instruction. Colonies were selected by treatment with 5 μg mL^−1^ puromycin (Santa Cruz Biotechnology) for 7 days.

### Isolation of Apoptotic bodies

Apoptotic bodies (ABs) were isolated as described previously (Proudfoot *et al*., 2000). After incubation for 48 h, media were collected and centrifuged at 2500 *g* for 8 min for ABs isolation.

### Preparation of AB‐free conditioned media

Cells were treated with oxLDL for 48 h, washed with PBS twice, and then changed to fresh media incubation for another 12 h. The media were collected as the conditioned media. For AB‐free conditioned media, the conditioned media were further collected and centrifuged at 2500 *g* for 8 min for ABs depletion.

### Western blot analysis

Cell lysates were subjected to Western blot analysis, as described previously (Cai *et al*., 2013). The intensity (area × density) of the individual bands on Bands was quantified by densitometry using Quantity One Software (Bio‐Rad, Hercules, CA).

### Determination of ALP activity

Alkaline phosphatase activity was measured by use of ALP assay kit (Wako, Japan) according to the manufacturer's instructions. ALP activity was normalized to levels of total protein.

### DiI‐oxLDL uptake

To measure DiI LDL uptake, VIC were plated in 12‐well plates in DMEM + 1% BSA. After overnight culture, VIC were incubated in the dark with DiI‐oxLDL (5 μg mL^−1^) (Yiyuan Biotechnology, Guangzhou, China) for 4 h on 37 °C. After the incubation, cells were washed 3× with PBS, detached with Versene, harvested with 1% BSA in HBSS, and subjected to flow cytometry analysis as previously described (Gabunia *et al*., 2017). The mean of DiI‐oxLDL fluorescence intensity was obtained from 10 000 cells. Data were calibrated to the protein concentration.

### Alizarin red staining

For analysis of osteoblastic differentiation in VICs, cells were incubated with indicated interventions in a conditioning medium (growth medium supplemented with 10 mmol L^−1^ β‐glycerophosphate, 10 nmol L^−1^ dexamethasone, 4 μg mL^−1^ cholecalciferol, and 8 mmol L^−1^ CaCl_2_) for 14 days as reported previously (Cai *et al*., 2016; Li *et al*., 2017). Media were changed every 3 days.

Alizarin red staining for calcium deposits was performed as described (Yang *et al*., 2009). Briefly, cell monolayers were washed twice with PBS and fixed for 15 min in 4% paraformaldehyde, followed by incubation with 0.2% alizarin red solution (pH 4.2) for 30 min. Excessive dye was removed by washing with distilled water. To quantitatively analyze alizarin red stain, wells were rinsed with distilled water, and alizarin red stains were bleached with 10% acetic acid at 85 °C. Supernatant was spectrophotometrically analyzed at 450 nm (Yang *et al*., 2009).

### Statistical analysis

Quantitative values are expressed as the mean ± SD and represent data from at least three independent experiments. After confirming that all variables were normally distributed by the Kolmogorov–Smirnov test followed by Q–Q plots analysis, statistical differences were determined by Student's *t*‐test for comparison between two groups and ANOVA followed by Bonferroni's multiple comparison test for comparison among three or more groups. *P* values of < 0.05 were considered statistically significant. All statistical calculations were carried out using SPSS version 17.0 (SPSS Inc, Chicago, IL, USA).

## Conflict of interest

None declared.

## Funding

This study was supported by the National Natural Science Foundation of China (81670390, 81300173, 81670351, 81501298, 81470384, and 81670259).

## Author contributions

The authors have made the following declarations about their contributions: Zhejun Cai, Meixiang Xiang, and Fei Li conceived and designed the experiments. Zhejun Cai, Baoqing Liu, Jia Wei, Zurong Fu, Yidong Wang, and Yaping Wang performed the experiments. Jian Shen, Liangliang Jia, Shengan Su, Xiaoya Wang, Xiaoping Lin, Han Chen, and Jian'an Wang analyzed the data and contributed reagents/materials/analysis tools. Zhejun Cai and Fei Li wrote the manuscript.

## Supporting information


**Fig. S1** Immunofluorescence staining of CHOP in AoV leaflets.
**Fig. S2** CHOP deficiency reduces transvalvular peak jet velocity and aortic leaflet thickness in Apoe^−/−^ mice.
**Fig. S3** CHOP deficiency suppresses apoptosis in Apoe^−/−^ mice.
**Fig. S4** CHOP deficiency suppresses pro‐osteogenic genes expression in AoV leaflets of Apoe^−/−^ mice.
**Fig. S5** Lentiviral CHOP shRNA transfection successfully suppressed CHOP expression.
**Fig. S6** CHOP deficiency does not affect oxLDL uptake in VIC.
**Fig. S7** Apoptotic bodies promote osteoblastic differentiation of VIC.
**Table S1** Measures of echocardiographic parameters of mice fed with western diet for 24 weeks.
**Table S2** Metabolic parameters of mice treated with western diet for 24 weeks.
**Table S3** Primers for quantitative real‐time PCR.
**Table S4** Clinical characteristics of patients with normal valves collected.
