# Supplementary material for: Deficiency of CCAAT/enhancer‐binding protein homologous protein (CHOP) prevents diet‐induced aortic valve calcification in vivo
Source: Aging Cell. 2017 Sep 10;16(6):1334–41. doi: 10.1111/acel.12674 (PMC5676062; doi:10.1111/acel.12674)
Supplement: Supplementary file 1 — Fig. S1 Immunofluorescence staining of CHOP in AoV leaflets. Fig. S2 CHOP deficiency reduces transvalvular peak jet velocity and aortic leaflet thickness in Apoe−/− mice. Fig. S3 CHOP deficiency suppresses apoptosis in Apoe−/− mice. Fig. S4 CHOP deficiency suppresses pro‐osteogenic genes expression in AoV leaflets of Apoe−/− mice. Fig. S5 Lentiviral CHOP shRNA transfection successfully suppressed CHOP expression. Fig. S6 CHOP deficiency does not affect oxLDL uptake in VIC. Fig. S7 Apoptotic bodies promote osteoblastic differentiation of VIC. Table S1 Measures of echocardiographic parameters of mice fed with western diet for 24 weeks. Table S2 Metabolic parameters of mice treated with western diet for 24 weeks. Table S3 Primers for quantitative real‐time PCR. Table S4 Clinical characteristics of patients with normal valves collected. [file ACEL-16-1334-s001.pdf]

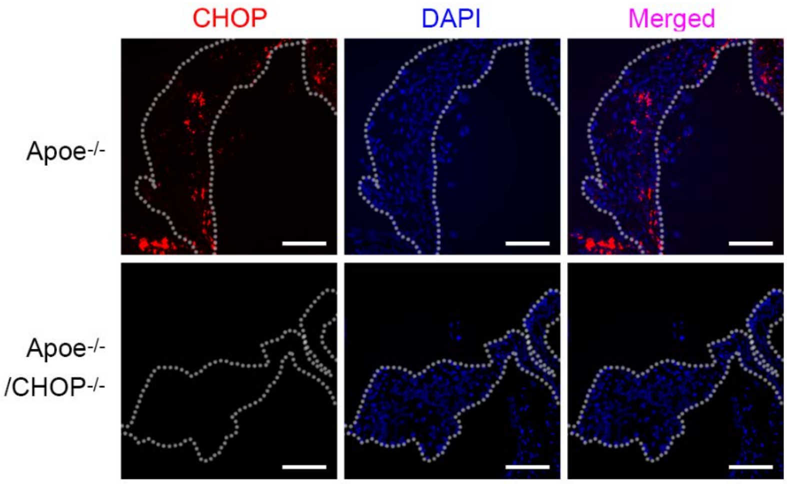

Supplemental Figure 1. Immunofluorescence staining of CHOP in AoV leaflets.

CHOP deficiency successfully deleted CHOP induction in AoV leaflets in Apoe$^{-/-}$ mice. (Scale bar: 100μm)

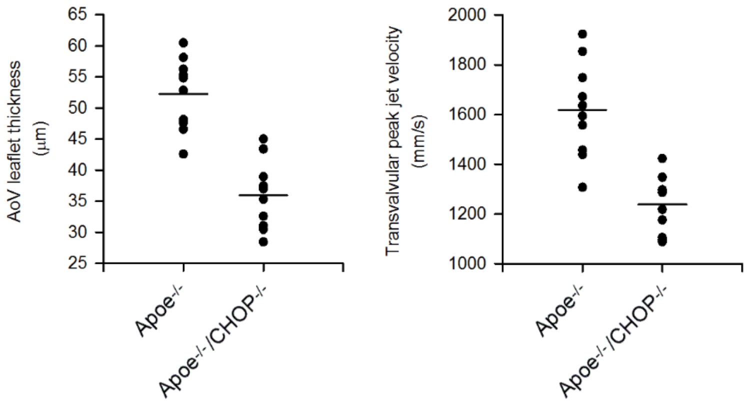

Supplemental Figure 2. CHOP deficiency reduces transvalvular peak jet velocity and aortic leaflet thickness in Apoe$^{-/-}$ mice.

After 24-week western diet treatment, CHOP deficiency in Apoe-/- mice significantly reduced AoV leaflet thickness and transvalvular peak jet velocity compared with Apoe$^{-/-}$ mice. (n=10 for each group. **P*<0.05)

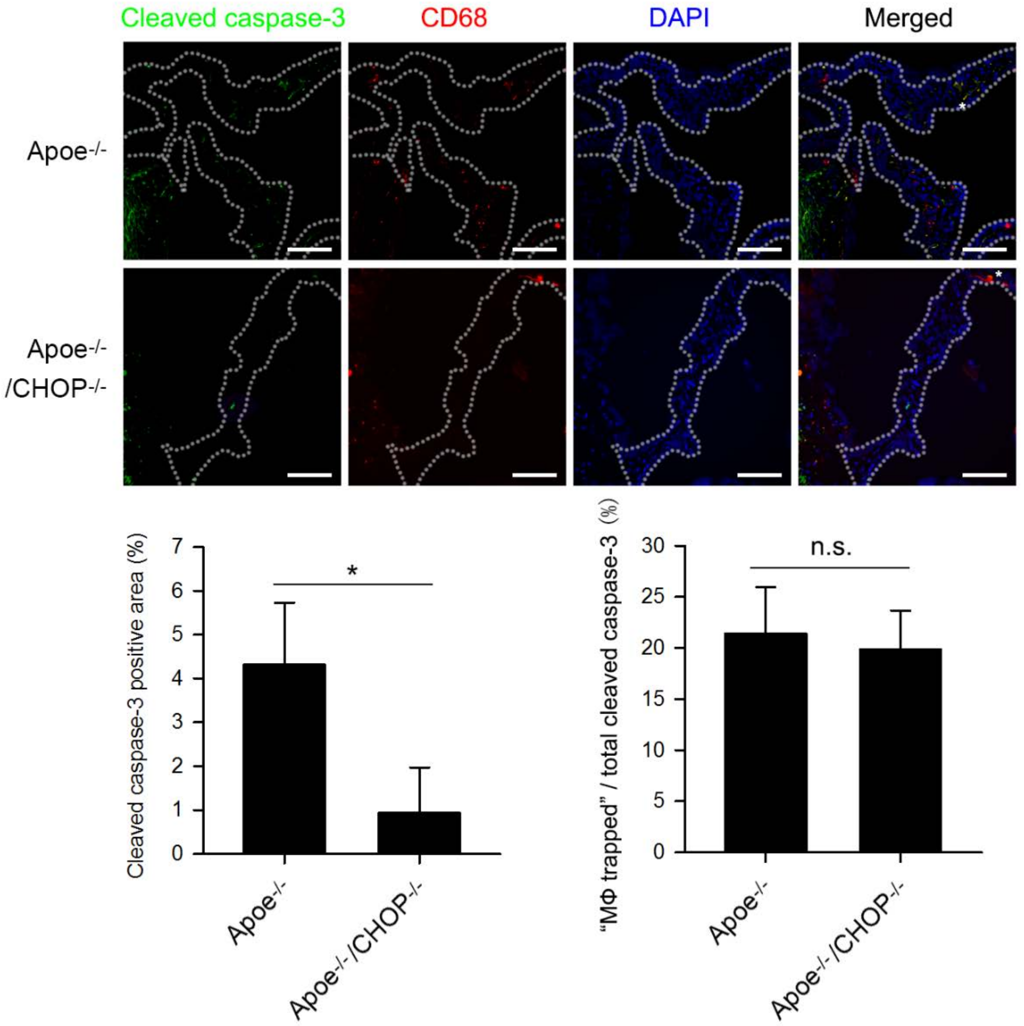

Supplemental Figure 3. CHOP deficiency suppresses apoptosis in Apoe$^{-/-}$ mice.

Immunofluorescence staining of cleaved caspase-3 (green) and macrophages marker CD68 (red) in AoV leaflets. After 24-week western diet treatment, CHOP deficiency significantly reduced cleaved caspase-3 staining in AoV leaflets in Apoe$^{-/-}$ mice. The ratio of macrophages "trapped" (white asterisk), which was CD68 dual stained, over total cleaved caspase-3 had no significant difference between Apoe$^{-/-}$ and Apoe$^{-/-}$CHOP$^{-/-}$ in AoV leaflets. (Scale bar: 100μm; n=10 for each group; *P<0.05; n.s. no significant difference.)

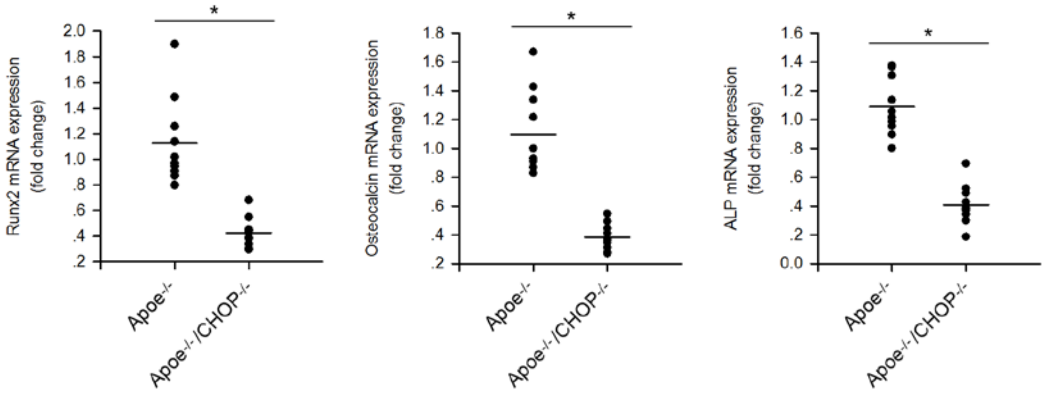

Supplemental Figure 4. CHOP deficiency suppresses pro-osteogenic genes expression in AoV leaflets of Apoe$^{-/-}$ mice.

Relative mRNA expression of Runx2, osteocalcin, and ALP in AoV leaflets of mice. Compared with Apoe$^{-/-}$ mice, all these genes were down-regulated in Apoe$^{-/-}$CHOP$^{-/-}$ mice. β-actin serves as internal reference. (n=10 for each group. *P<0.05)

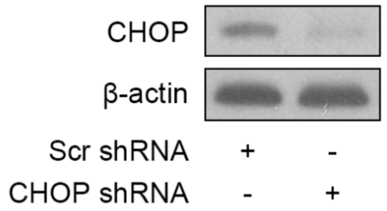

Supplemental Figure 5. Lentiviral CHOP shRNA transfection successfully suppressed CHOP expression.

Western blot analysis showed that after 14 days of lentiviral CHOP shRNA transfection, CHOP expression was markedly suppressed.

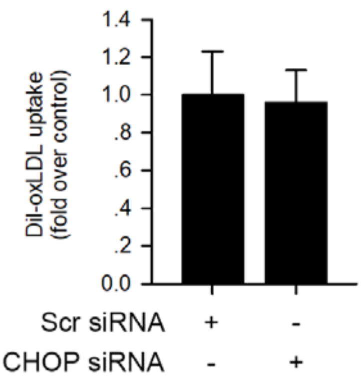

Supplemental Figure 6. CHOP deficiency does not affect oxLDL uptake in VIC.

After transfection of VIC with scrambled or CHOP siRNA, cells were incubated with Dil-oxLDL for 4 h. CHOP silencing did not alter uptake of Dil-oxLDL in VIC.

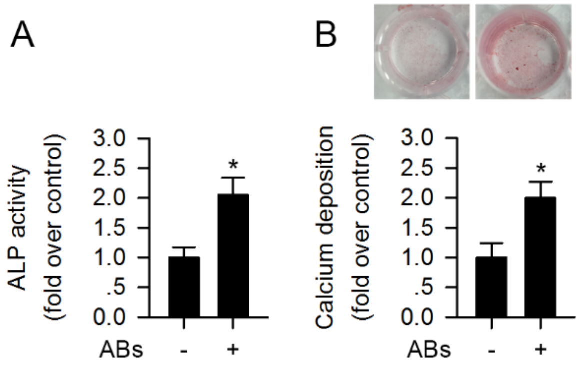

Supplemental Figure 7. Apoptotic bodies promote osteoblastic differentiation of VIC.

A. Apoptotic bodies (ABs) significantly increased ALP activity of VIC. **B.** Alizarin red staining of calcium nodules of VIC. ABs markedly promoted calcium deposits of VIC. (n=3 for each experiment; *P<0.05 vs control)

Supplemental Table 1. Measures of echocardiographic parameters of mice fed with western diet for 24 weeks

Echocardiography	Apoe$^{-/-}$	Apoe$^{-/-}$CHOP$^{-/-}$
Heart rate, bpm	443 ± 18	438 ± 22
CO, mL/min	34.5 ± 2.2	33.7 ± 2.4
LV mass, mg	224.2 ± 4.3	226.1 ± 5.5
LVIDd, mm	4.11 ± 0.12	4.14 ± 0.09
FS, %	34.1 ± 1.1	33.7 ± 0.9
EF, %	71.5 ± 1.8	70.9 ± 1.6

N=10 for each group.

CO, cardiac output; LV, left ventricular; LVIDd, LV interior diameter in end-diastole; FS, fractional shortening; EF, ejection fraction.

Supplemental Table 2. Metabolic parameters of mice treated with western diet for 24 weeks

	$Apoe^{-/-}$	$Apoe^{-/-}CHOP^{-/-}$
Body weight (g)	33.78±0.52	33.24±0.47
Triglyceride (mg/dL)	137.84±14.07	132.09±13.02
Total cholesterol (mg/dL)	1203.57±80.72	1134.13±70.35
Blood glucose (mg/dL)	152.05±13.76	147.27±16.88
Calcium (mg/dL)	8.68±0.25	8.78±0.32
Phosphorus (mg/dL)	3.53±0.37	3.46±0.31

N=10 for each group.

Supplemental Table 3. Primers for quantitative real-time PCR

Genes	Primer sequence
Runx2	F-5’-AGAGTCAGATTACAGATCCCAGG-3’<br>R-5’-AGGAGGGGTAAGACTGGTCATA-3’
ALP	F-5’-TTGTGCCAGAGAAAGAGAGAGA-3’<br>R-5’-GTTTCAGGGCATTTTTCAAGGT-3’
Osteocalcin	F-5’-CTCTCTCTGCTCACTCTGCT-3’<br>R-5’-TTTGTAGGCGGTCTTCAAGC-3’
β-actin	F-5’-CGCCACCAGTTCGCCATGGA-3’<br>R-5’-TACAGCCCGGGGAGCATCGT-3’

Supplemental Table 4. Clinical characteristics of patients with normal valves collected

**Patients No.**	1	2	3
Age (years)	51	52	55
Gender	Male	Male	Male
Body mass index (kg/m^2)	21.4	19.3	20.7
Hypertension	no	no	no
Hypercholesterolemia	yes	no	no
Diabetes mellitus	no	no	no
Smoking	yes	yes	yes
Transvalvular gradient (mmHg)	16.4	13.9	18.5
Diagnosis	DCM	DCM	DCM

DCM: dilated cardiomyopathy.
